# Analysis of the distribution pattern of the ectomycorrhizal fungus *Cenococcum geophilum* under climate change using the optimized MaxEnt model

**DOI:** 10.1002/ece3.10565

**Published:** 2023-09-25

**Authors:** Yexu Zheng, Chao Yuan, Norihisa Matsushita, Chunlan Lian, Qifang Geng

**Affiliations:** ^1^ College of Forestry Shandong Agricultural University Tai'an China; ^2^ College of Grassland Science and Technology China Agricultural University Beijing China; ^3^ College of Forestry Fujian Agriculture and Forestry University Fuzhou China; ^4^ Graduate School of Agricultural and Life Sciences The University of Tokyo Tokyo Japan; ^5^ Asian Research Center for Bioresource and Environmental Sciences, Graduate School of Agricultural and Life Sciences The University of Tokyo Nishitokyo‐shi Tokyo Japan

**Keywords:** *Cenococcum geophilum*, climate change, kuenm, MaxEnt model, migration prediction

## Abstract

*Cenococcum geophilum* (*C. geophilum*) is a widely distributed ectomycorrhizal fungus that plays a crucial role in forest ecosystems worldwide. However, the specific ecological factors influencing its global distribution and how climate change will affect its range are still relatively unknown. In this study, we used the MaxEnt model optimized with the kuenm package to simulate changes in the distribution pattern of *C. geophilum* from the Last Glacial Maximum to the future based on 164 global distribution records and 17 environmental variables and investigated the key environmental factors influencing its distribution. We employed the optimal parameter combination of RM = 4 and FC = QPH, resulting in a highly accurate predictive model. Our study clearly shows that the mean temperature of the coldest quarter and annual precipitation are the key environmental factors influencing the suitable habitats of *C. geophilum*. Currently, appropriate habitats of *C. geophilum* are mainly distributed in eastern Asia, west‐central Europe, the western seaboard and eastern regions of North America, and southeastern Australia, covering a total area of approximately 36,578,300 km^2^ globally. During the Last Glacial Maximum and the mid‐Holocene, *C. geophilum* had a much smaller distribution area, being mainly concentrated in the Qinling‐Huaihe Line region of China and eastern Peninsular Malaysia. As global warming continues, the future suitable habitat for *C. geophilum* is projected to shift northward, leading to an expected expansion of the suitable area from 9.21% to 21.02%. This study provides a theoretical foundation for global conservation efforts and biogeographic understanding of *C. geophilum*, offering new insights into its distribution patterns and evolutionary trends.

## Graphical abstract

MaxEnt Modeling was used to predict the distribution of *Cenococcum geophilum*. *C. geophilum* mainly occurs in East Asia, Europe, and North America. Temperature and precipitation were critical to the distribution of *C. geophilum*. The habitat of *C. geophilum* is projected to shift northward under future climate scenarios. New insights have been provided into *C. geophilum* distribution pattern formation and evolution.

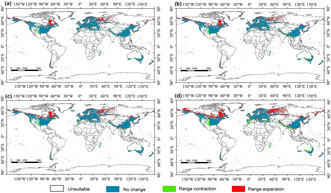

## 1 INTRODUCTION

The severity of climate extremes is increasing, and recent and projected climate change is exceeding historical trends (Parmesan et al., 2022; Pörtner et al., 2022). This exceeds the potential for species to adapt, necessitating range shifts for many species to survive (Donelson et al., 2023; Scheffers et al., 2016). Many studies suggest that the migratory success of plant species under climate change may depend on their mycorrhizal fungal partners (Bennett & Classen, 2020; Wurzburger et al., 2017). Ectomycorrhizal (ECM) interactions are the dominant mycorrhizal symbiosis for plants in temperate and boreal forests, and there is evidence that the absence of ECM fungi limits plant dispersal (Nuñez et al., 2009; Tedersoo et al., 2012). Therefore, clarifying the dynamics of geographic distribution of ECM fungi under climate change is critical for predicting the impact of climate warming on forest ecosystems globally.

Climate change impacts ECM fungi in diverse ways, making it challenging to predict how it will affect their distributions and activities (Meineke et al., 2018). Species distribution models (SDMs) can predict fungal species distributions and provide a new approach for studying the response of ECM fungi to climate change (Borgelt et al., 2022; Briscoe et al., 2023; Ponti & Sannolo, 2023). These models relate species presence data to climatic and topographic variables, enabling estimates of the past, present, and future distribution of target species (Buonincontri et al., 2023; Chowdhury, 2023; Riva et al., 2023). Among SDMs, the MaxEnt model based on the principle of maximum entropy, has proven most effective in simulating the geographical distribution of species (Phillips et al., 2006; Phillips & Dudík, 2008; West et al., 2016). The outstanding advantage of the MaxEnt model is its reliable prediction accuracy even with limited sample size and insufficient data (Shi et al., 2023; Zhou et al., 2023). Many studies have demonstrated the reliability of MaxEnt in predicting the potential distribution of fungi (Banasiak et al., 2019; Cogliati et al., 2023; Wei et al., 2021).

ECM fungi play an essential role in nutrient cycling and ecosystem functioning in temperate forest ecosystems (Read et al., 2004). ECM fungi cover the finest root branches of trees and provide nutrients from the soil in exchange for carbon synthesized by the plant (Nehls & Plassard, 2018). In boreal forest soils, ECM fungi contribute up to 39% of microbial biomass (Smith & Read, 2010). Studies have shown that the species richness of ECM fungi follows an unimodal relationship with latitude, peaking between 4000 and 4500 km from the equator (Tedersoo et al., 2012, 2014). Additionally, ECM fungi exhibit changes along the elevation gradient (Bahram et al., 2012; Shigyo & Hirao, 2021). These diversity gradients are largely influenced by climatic variables, particularly mean annual temperature and precipitation (Looney et al., 2016). The physical and chemical properties of the soil, such as pH and organic matter content, also strongly influence the ECM fungal community (Ge et al., 2017). Despite these efforts, the relative importance of different ecological factors to ECM fungal communities and how future climate change will affect the distribution of ECM fungi globally remain relatively unknown.

*Cenococcum geophilum*, a globally distributed ECM fungus, is considered one of the most abundant and ubiquitous species in forest soils and on woody plant roots (Trappe, 1962). *C. geophilum* occupies a wide range of ecological niches, from tundra to rainforests, and forms symbiotic relationships with over 200 tree species (Dauphin et al., 2021; Obase et al., 2017). Therefore, this fungal group could serve as a model system for studying the biogeography of ECM fungi, and its dynamics under future climate change scenarios may reflect the distribution dynamics of the majority of forest tree species. However, current research on *C. geophilum* mainly focuses on its genetic phylogeny and environmental tolerance (Li et al., 2022; Obase et al., 2017; Peter et al., 2016), and there have been no reports on the use of species distribution models to predict the global distribution dynamics of *C. geophilum* under climate change.

We hypothesize that climatic factors are the main drivers of the distribution of *C. geophilum* at a global scale. As global temperature increases, we predict that low latitudes will reach the limiting temperature suitable for the distribution of *C. geophilum* in the future. Higher latitudes will provide survival refuges for *C. geophilum*, and suitable distribution areas are expected to move to higher latitudes. In order to scientifically explore the response of the potential geographical distribution of ECM fungi to climate change in different periods, this study aims to model the geographical distribution of the species *C. geophilum* using the MaxEnt model. Our study focuses on three key objectives: (1) reconstructing the historical changes in the geographic distribution patterns of *C. geophilum* since the Last Glacial Maximum period; (2) analyzing the relationship between the predicted potential distribution area of *C. geophilum* and the main environmental factors; and (3) providing new insights into the conservation and utilization of *C. geophilum* as well as other ECM fungi.

## 2 MATERIALS AND METHODS

### 2.1 Species occurrence data sources

**FIGURE 1:**
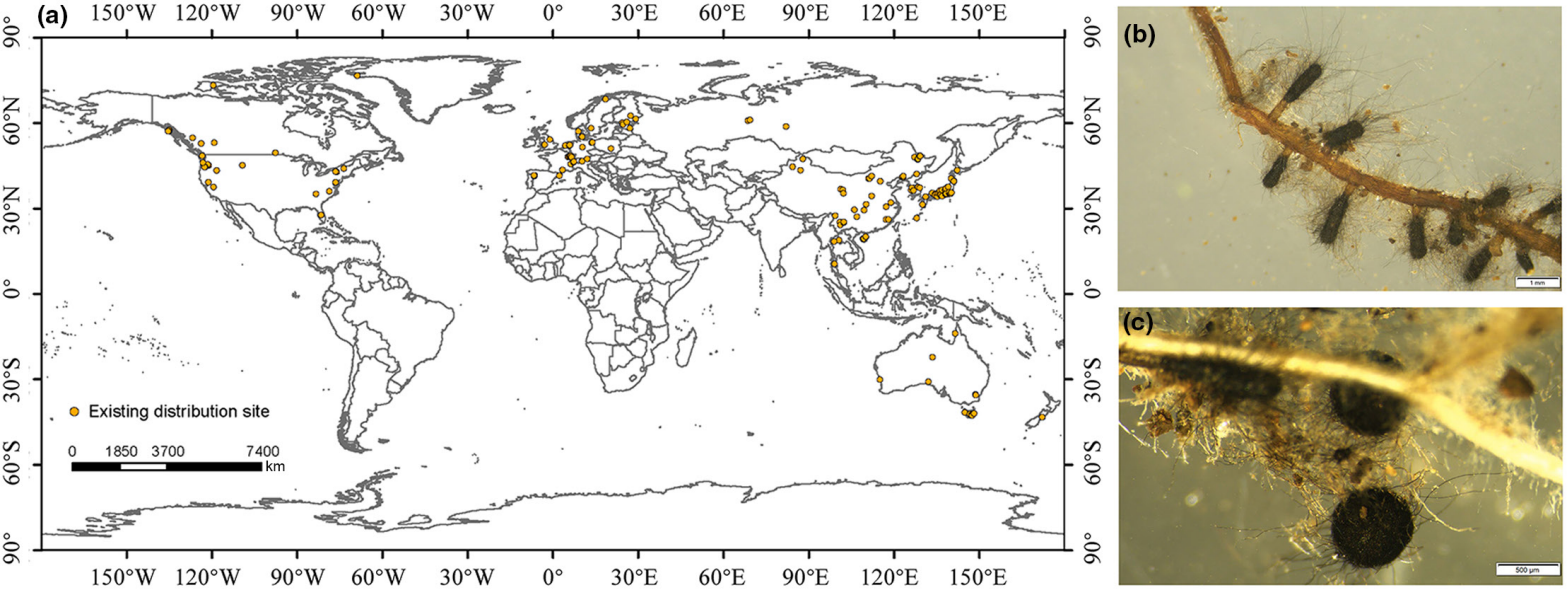
(a) Geographic locations of *Cenococcum geophilum* worldwide; (b) the mycorhiza of *C. geophilum* on the roots of *Pinus densiflora*; (c) sclerotium of *C. geophilum*.

In this study, we collected 203 occurrence records of *C. geophilum*, including 35 records obtained from fieldwork conducted in China and Japan and 168 records extracted from the Global Biodiversity Information Facility database (GBIF, https://www.gbif.org/). When geographic coordinates were unavailable occurrence data, we used Google Earth to determine latitude and longitude. To address issues of spatial autocorrelation and pseudo‐replication of species occurrence data impacting model outputs, we used ENMTools software to ensure that each 5 km^2^ grid cell contained only one distribution record (Naimi et al., 2011; Warren et al., 2010). Ultimately, we obtained 164 records representing the effective distribution of *C. geophilum* (Figure 1).

### 2.2 Environmental variable

We initially selected 36 environmental parameters that could potentially affect the distribution of *C. geophilum* in order to model species distribution patterns, including 19 bioclimatic variables, 16 soil variables, and 1 topographic variable (Table S1). We resampled the environmental variables at a spatial resolution of 2.5 min, which is approximately 5 km^2^. Data on bioclimatic variables for different time periods, including the Last Glacial Maximum (LGM), Middle Holocene (MH), current (1970–2000), and future (2050s, 2070s), were obtained from the Worldclim database (http://worldclim.org). The topographic variable data were downloaded from the WorldClim database, while the soil variable data were gathered from the Harmonized World Soil Database (HWSD, https://www.fao.org/soils‐portal/data‐hub/soil‐classification/en). For future climate scenarios, we used BCC CSM2‐MR climate change modeling data to predict the distribution of *C. geophilum* under two Shared Socioeconomic Pathways (SSPs): SSP 1–2.6 (2.6 Wm^−2^) and SSP 5–8.5 (5.8 Wm^−2^) at two time points (the 2050s and 2070s; O'Neill et al., 2016; Riahi et al., 2017). BCC CSM2‐MR is one of the most commonly used models for predicting the global climate response to increasing greenhouse gas emissions (Zhang et al., 2018).

Thirty‐six environmental variables were inputted into MaxEnt for preliminary modeling to calculate the contribution rates of each environmental factor. Variables with contribution rates below 0.10 were removed. It has been demonstrated that high collinearity among predictor variables can reduce the accuracy of the model's predictions (Zhao, Xiao, et al., 2022). Therefore, we used the ENMTools software to calculate the Pearson correlation coefficient (*r*) of the environmental variables (Figure S1) in order to identify highly correlated variables and minimize collinearity among the remaining environmental variables (Warren et al., 2010). For any pair of highly correlated environmental factors (*r* > .80; Bowen & Stevens, 2020), we retained the variable that had a greater contribution to the growth of *C. geophilum*. Finally, the model was established using seven climate variables, one topography variable, and nine soil variables (Table S2).

### 2.3 Modeling procedure and model evaluation

MaxEnt v3.4.1 was used to forecast potentially suitable habitats for *C. geophilum* based on species occurrence data and environmental variables (Gao et al., 2022; Zhao, Lei, et al., 2022). We randomly selected 70% of the *C. geophilum* occurrence records as training data and the remaining 30% as testing data (Liu & Shi, 2020). The maximum number of iterations was set at 5000 to ensure sufficient time for the model to converge.

To enhance model performance and prevent overfitting, we optimized Feature Combination (FC) and Regularization Multiplier (RM) using the “kuenm” package in the R software (Cobos et al., 2019; Phillips & Dudík, 2008). A total of 600 candidate models were generated by combining 40 RM settings (0.1–4.0 with intervals of 0.1) and 15 different FC (L, Q, P, H, LQ, LP, LH, QP, QH, PH, LQP, LQH, LPH, QPH, LQPH; where L = linear, Q = quadratic, P = product, H = hinge; Cobos et al., 2019; Phillips et al., 2017). The complexity and goodness‐of‐fit of these 600 models were evaluated based on a 5% omission rate and the delta from the Akaike information criterion (AICc; Cobos et al., 2019). AICc was used as the performance metric, and the model parameters of the candidate model with the smallest delta AICc were selected for modeling (Liu et al., 2018). Moreover, to reduce potential variation and randomness in the model simulation results, ten replicated rounds of cross‐validation were performed, and the final findings are the average of these ten repetitions.

To assess the overall performance of the MaxEnt model simulation results, the threshold‐independent analysis of the receiver operating characteristic (ROC) curve ws employed (Gong et al., 2022; Porfirio et al., 2014). However, it has been shown that AUC values are inadequate in evaluating models of the potential distribution of species, so we used TSS (true skill statistics) as a complementary (Ahmadi et al., 2023; Jiménez‐Valverde, 2012). The value of AUC ranges from 0 to 1 and the model performance is classified as failing (AUC: 0.5–0.6), poor (AUC: 0.6–0.7), fair (AUC: 0.7–0.8), good (AUC: 0.8–0.9), or excellent (AUC: 0.9–1.0; Adhikari et al., 2018). The value of TSS varies between −1 and +1, with values close to 1 indicating that the model is almost perfect (Allouche et al., 2006).

### 2.4 Potentially suitable area partitions

We used ArcGIS v10.6 to divide and visualize the layers representing potentially suitable habitats for *C. geophilum*. The habitat suitability was quantitatively assessed on a scale ranging from 0 to 1. We classified habitats as suitable or unsuitable for the survival of the target species based on a decision threshold of 10 percentile training logistic presence threshold (10% LPT), as described by Pearson et al. (2007). The MaxEnt model simulation results indicated that the 10% LPT was 0.2217. Therefore, the range of suitable habitats for *C. geophilum* was divided into four categories: unsuitable habitat (0.00–0.2217), poorly suitable habitat (0.2217–0.40), moderately suitable habitat (0.40–0.60), and highly suitable habitat (0.60–1.00). Furthermore, we analyzed the changes in spatial patterns of *C. geophilum* under future climate scenarios relative to its current range, identifying areas of range expansion, contraction, and no change.

## 3 RESULTS

### 3.1 Model accuracy evaluation for simulating the potentially suitable habitats of *C. geophilum*

Based on the 164 distribution points of *C. geophilum* and 17 environmental variables, we simulated the potential global distribution of *C. geophilum* at different periods using the MaxEnt model. The default parameters of the model were RM = 1 and FC = LQPH. Through optimization, the parameters of the model were changed to RM = 4, FC = QPH, and delta AICc = 0 (Table 1), which indicated the model's optimality. Additionally, the model's omission rate was reduced by 74.9% compared to the preoptimization period. These results showed that the optimized parameter combination significantly reduces the complexity and overfitting of the model. Therefore, RM = 4 and FC = QPH were finally chosen as the parameter settings for 10 replications of the modeling. After testing, the average AUC value of 10 repetitions of modeling was 0.878 (Figure S2) and the average TSS value was 0.959 (Table S3), indicating that the optimized MaxEnt model achieved good results in predicting the potential distribution of *C. geophilum*.

**TABLE 1:** Evaluation metrics of the default and optimal Maxent models using the kuenm package.

Setting	FC	RM	Omission_rate_at_5%	AICc	Delta_AICc
Default	LQPH	1	0.108108	4504.867	504.4341
Optimized	QPH	4	0.027027	4000.433	0

### 3.2 Evaluation of environmental factors for the potentially suitable habitats of *C. geophilum*

The important environmental factors influencing the potentially suitable habitats of *C. geophilum* were confirmed using the percentage contribution and Jackknife test results. For *C. geophilum*, mean temperature of the coldest quarter (Bio11, 38.9%) and annual precipitation (Bio12, 24.5%) were the two environmental variables with the highest percentage contribution (Table S2). Two factors, temperature seasonality (Bio4, 8.6%) and topsoil electrical conductivity (T_ECE, 6.7%), also had relatively high percentage contributions (Table S2). The Jackknife test revealed that mean temperature of the coldest quarter (Bio11), annual precipitation (Bio12), temperature seasonality (Bio4), and precipitation of the driest quarter (Bio17, 4%) ranked highest in terms of the regularized training gain when modeled using a single environmental factor (Figure S3). Comprehensive Jackknife test and percentage contribution analysis showed that bio11, bio12, bio4, bio17, and T_ECE were the dominant environmental factors affecting the distribution of *C. geophilum* under current climatic conditions. The cumulative contribution of these five factors accounted for 82.70% of the total contribution. These findings suggest that climatic factors, particularly Bio11 and Bio12, are key in determining the ecological niche of *C. geophilum*. In contrast, soil factors have limited influence on its ecological niche, with only T_ECE showing some influence.

**FIGURE 2:**
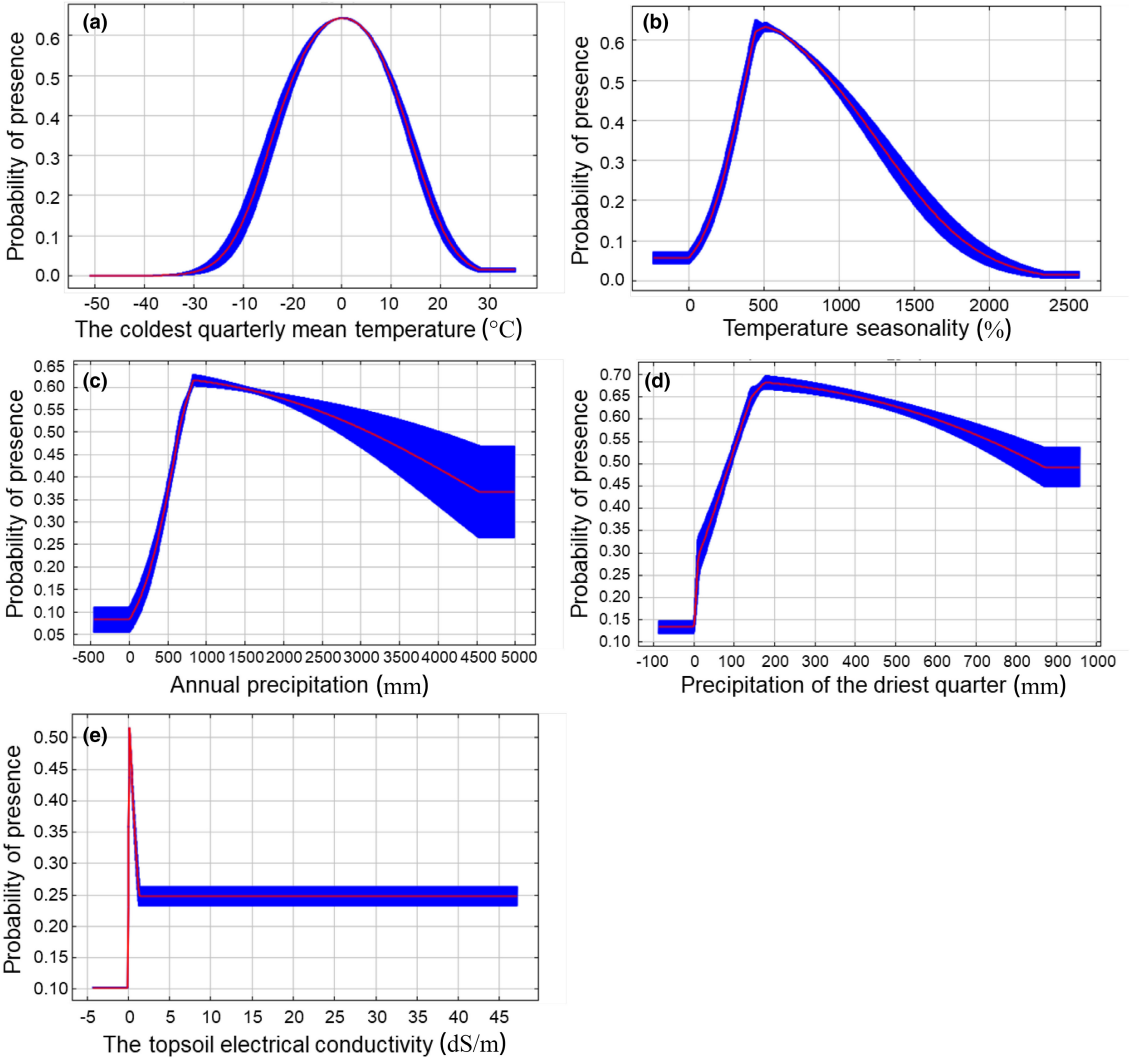
Relationships between key environmental variables and the probability of existence for *Cenococcum geophilum*.

To further clarify the climatic characteristics of potentially suitable areas for *C. geophilum*, response curves were plotted to illustrate how the logistic predictions of habitat suitability varied with changes in five important environmental variables (Figure 2). We found that all five important environmental variables showed a single‐peak curve relationship with the probability of the presence of *C. geophilum*. In other words, the probability of the presence of *C. geophilum* gradually increased with increasing values of these five environmental factor variables within a certain range, and after reaching the peak value, the probability of its presence gradually decreased with increasing values of the variable. Using the 10% LPT (0.2217) as the threshold value, the range of key environmental factors influencing the distribution of *C. geophilum* was evaluated. The suitable range for Bio11 was approximately −17.92 to 17.42°C, with an optimal value of around 0.05°C. The suitable range for Bio4 was approximately 188.54%–1548.8%, with an optimal value of about 510.95%. Bio12 had a suitable range greater than 280.73 mm, and an optimal value of 840.40 mm. Bio17 had a suitable range greater than 4.87 mm, with an optimum value of 178.18 mm. T_ECE had a suitable range greater than 0.034 dS/m, and an optimal value of 0.14 dS/m.

### 3.3 Global distribution of suitable habitats for *C. geophilum* under current climatic conditions

**FIGURE 3:**
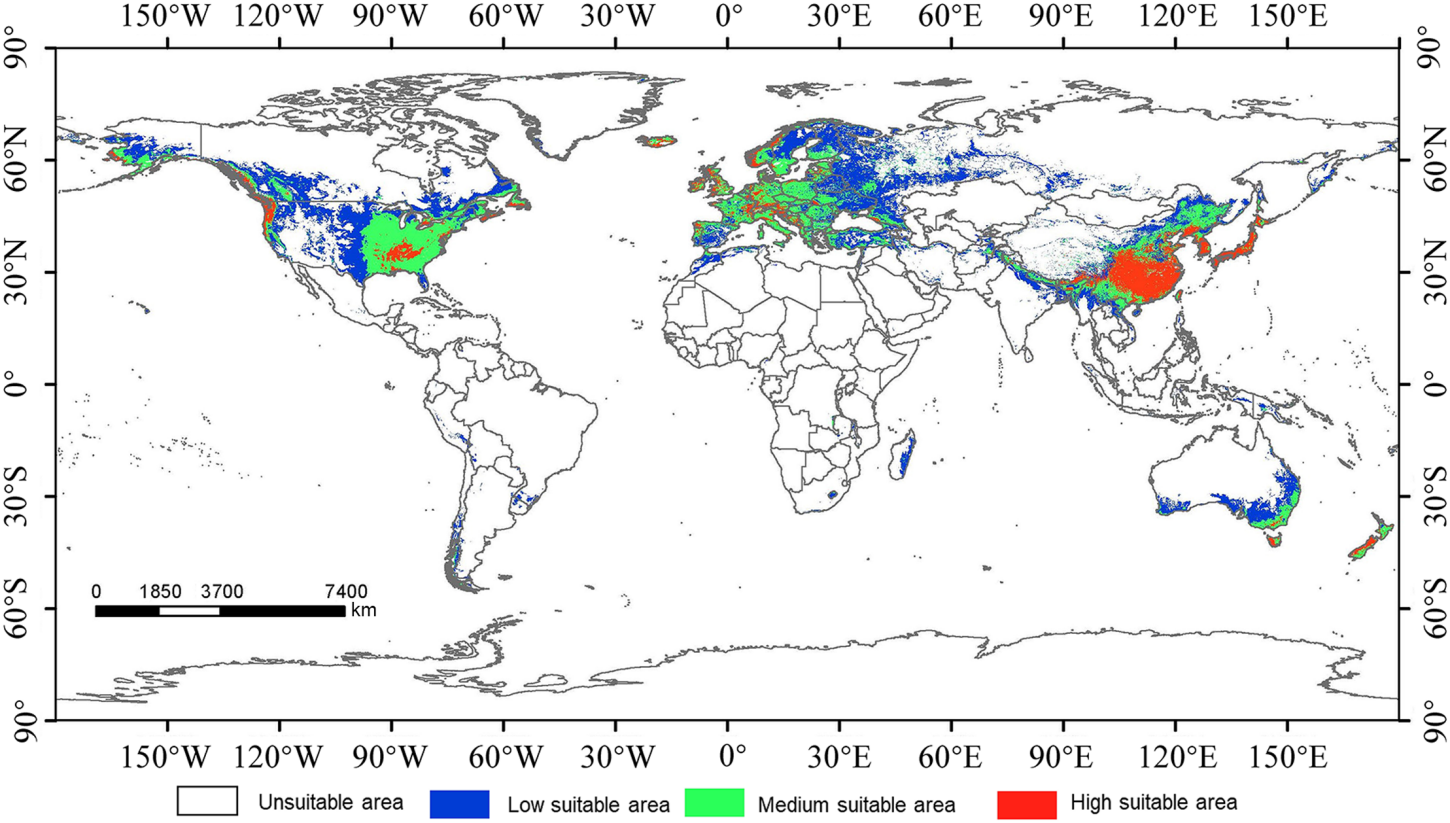
Potentially suitable areas for *Cenococcum geophilum* under current climate conditions worldwide. The red areas represent high suitability areas, the green areas represent medium suitability areas, the blue areas represent low suitability areas, and the white areas represent unsuitability areas.

Under current climatic conditions, suitable habitats for *C. geophilum* in the Northern Hemisphere are concentrated in East Asia, west‐central Europe, the western seaboard, and eastern regions of North America (Figure 3). In the southern hemisphere, the suitable survival zone for *C. geophilum* is small, mainly concentrated in southeastern Australia (Figure 3). The worldwide area of suitable habitats for *C. geophilum* is approximately 36,578,300 km^2^, with a highly suitable habitat area of 5,999,700 km^2^ (Figure 3). The highly suitable habitats for *C. geophilum* are mainly found in southern and northern regions of China, southern Korea, and Japan in Asia; western England, eastern France, southern Germany, Switzerland, Ireland, the western coast of Norway, central and eastern Iceland, northwestern Spain, northern Portugal, and central Italy in Europe; the central Mississippi River and Gulf of Alaska coast in the eastern United States, the Gulf of Alaska coast in western Canada, and the island of Newfoundland in eastern Canada in North America.

### 3.4 Global distribution of suitable habitats for *C. geophilum* under the paleoclimate climatic conditions

**FIGURE 4:**
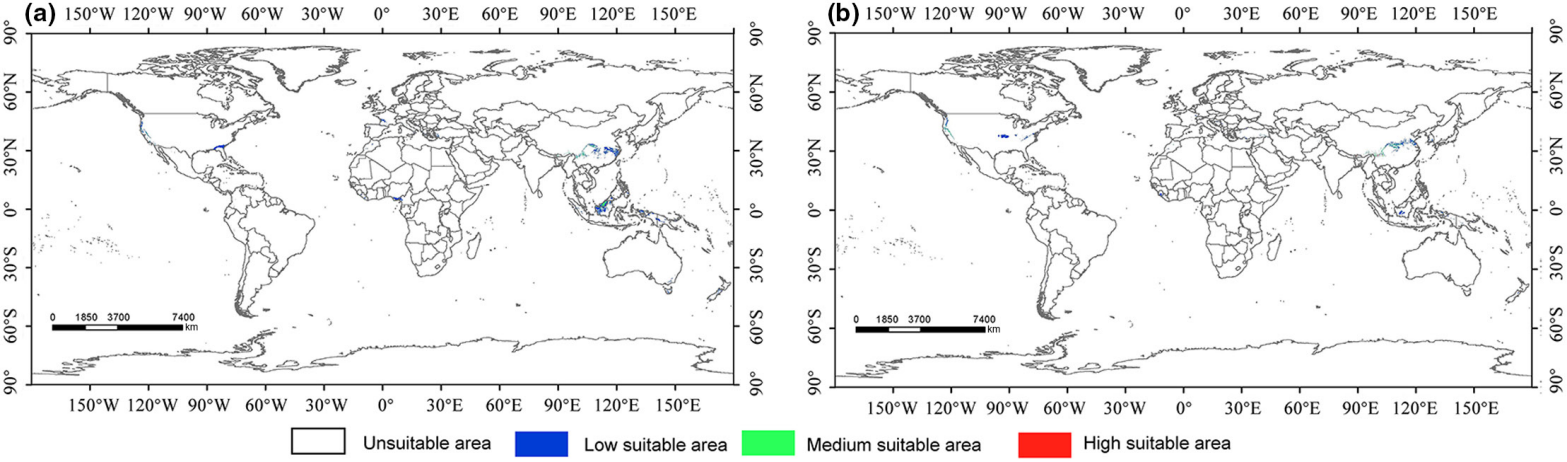
Potentially suitable areas for *Cenococcum geophilum* under paleoclimate change scenarios worldwide during the Last Glacial Maximum (LGM) and Mid Holocene (MH; a: LGM; b: MH).

The paleoclimate scenario had much fewer suitable areas for *C. geophilum* compared with the current conditions (Figure 4). During the Last Glacial Maximum (LGM), approximately 1,088,200 km^2^ of appropriate habitats existed for *C. geophilum*, representing only 2.97% of the currently suitable habitats (Figure 4). During this period, the suitable habitats for *C. geophilum* were concentrated around the vicinity of the Qinling‐Huaihe Line region of China, eastern Peninsular Malaysia, and the west coast of North America.

During the Mid‐Holocene (MH), appropriate habitats for *C. geophilum* were concentrated in the North of the Qinling‐Huaihe Line in China, the west coast of North America, and the middle Mississippi River (Figure 4). The overall suitable habitat area for *C. geophilum* was approximately 630,600 km^2^ (Figure 4). From the LGM to the MH, the suitable habitat area declined by 42.05%, while the overall suitable range migrated northward (Figure 4).

### 3.5 Global distribution of suitable habitats for *C. geophilum* under future climatic conditions

**FIGURE 5:**
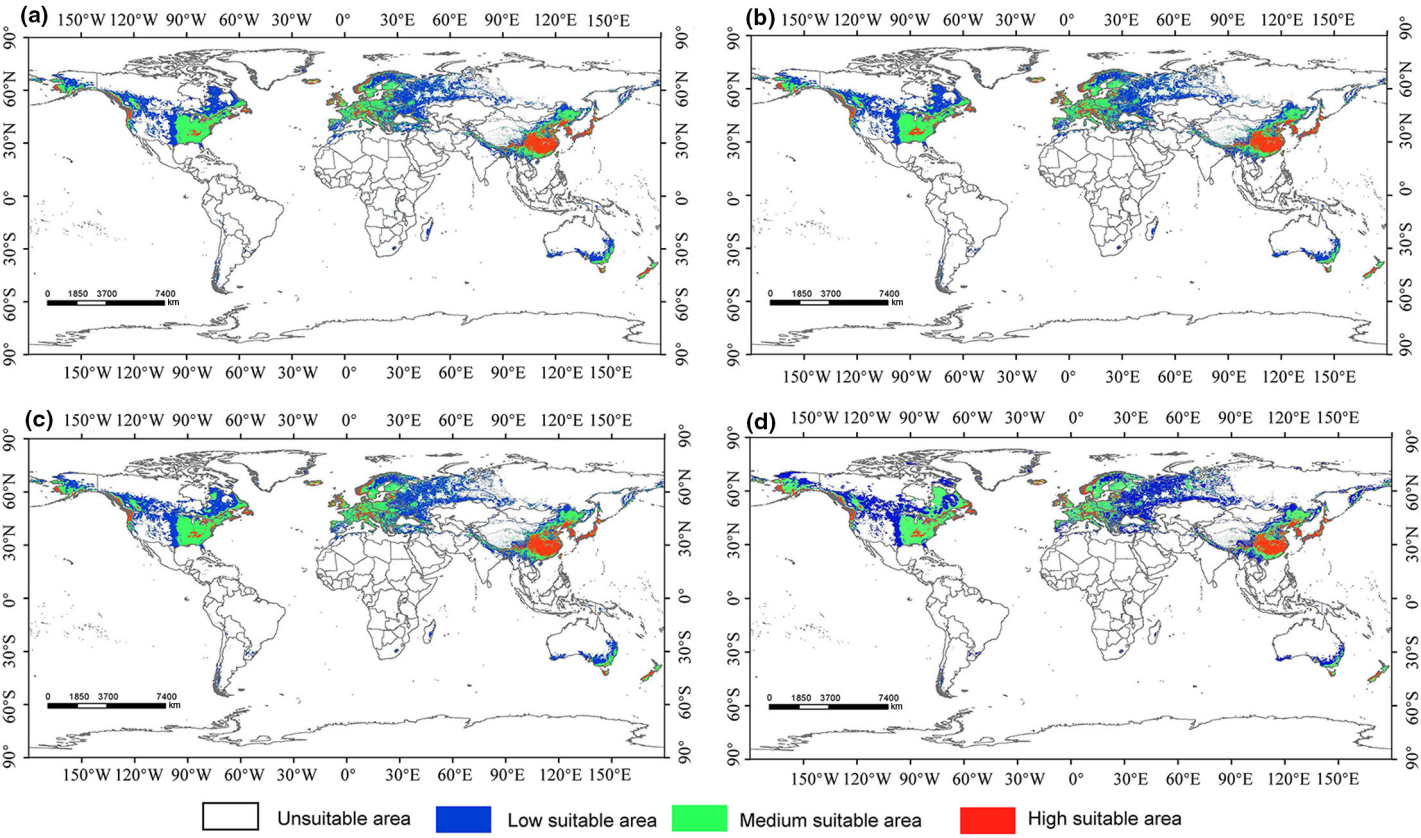
Potentially suitable areas for *Cenococcum geophilum* under future change scenarios worldwide in the 2050s and 2070s (a: 2050 SSP 1–2.6; b: 2070 SSP 1–2.6; c: 2050 SSP 5–8.5; d: 2070 SSP 5–8.5).

**FIGURE 6:**
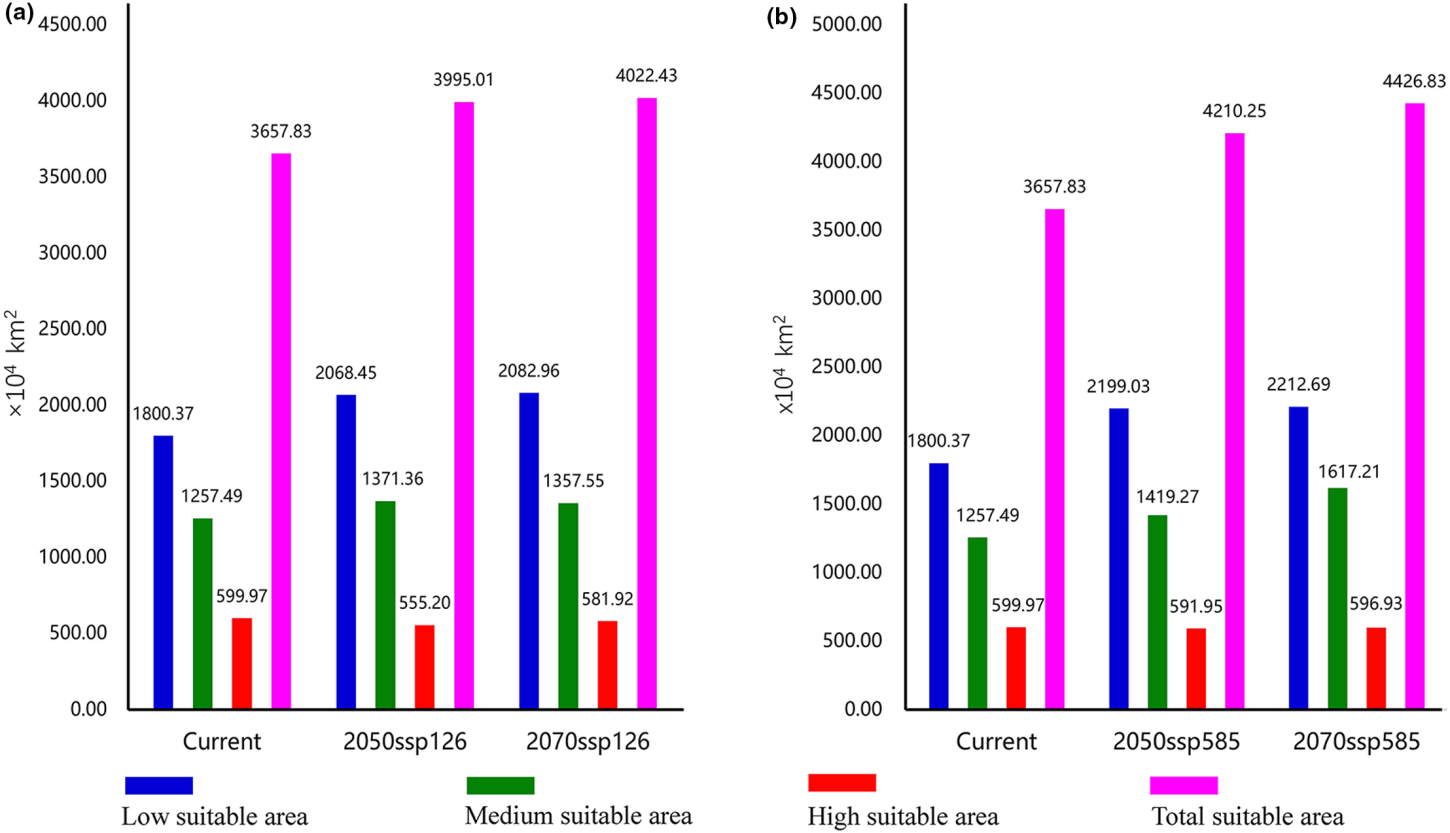
Areas with varying potential suitability for *Cenococcum geophilum* relative to the current situation under different climate scenarios worldwide (a: SSP 1–2.6; b: SSP 5–8.5).

**FIGURE 7:**
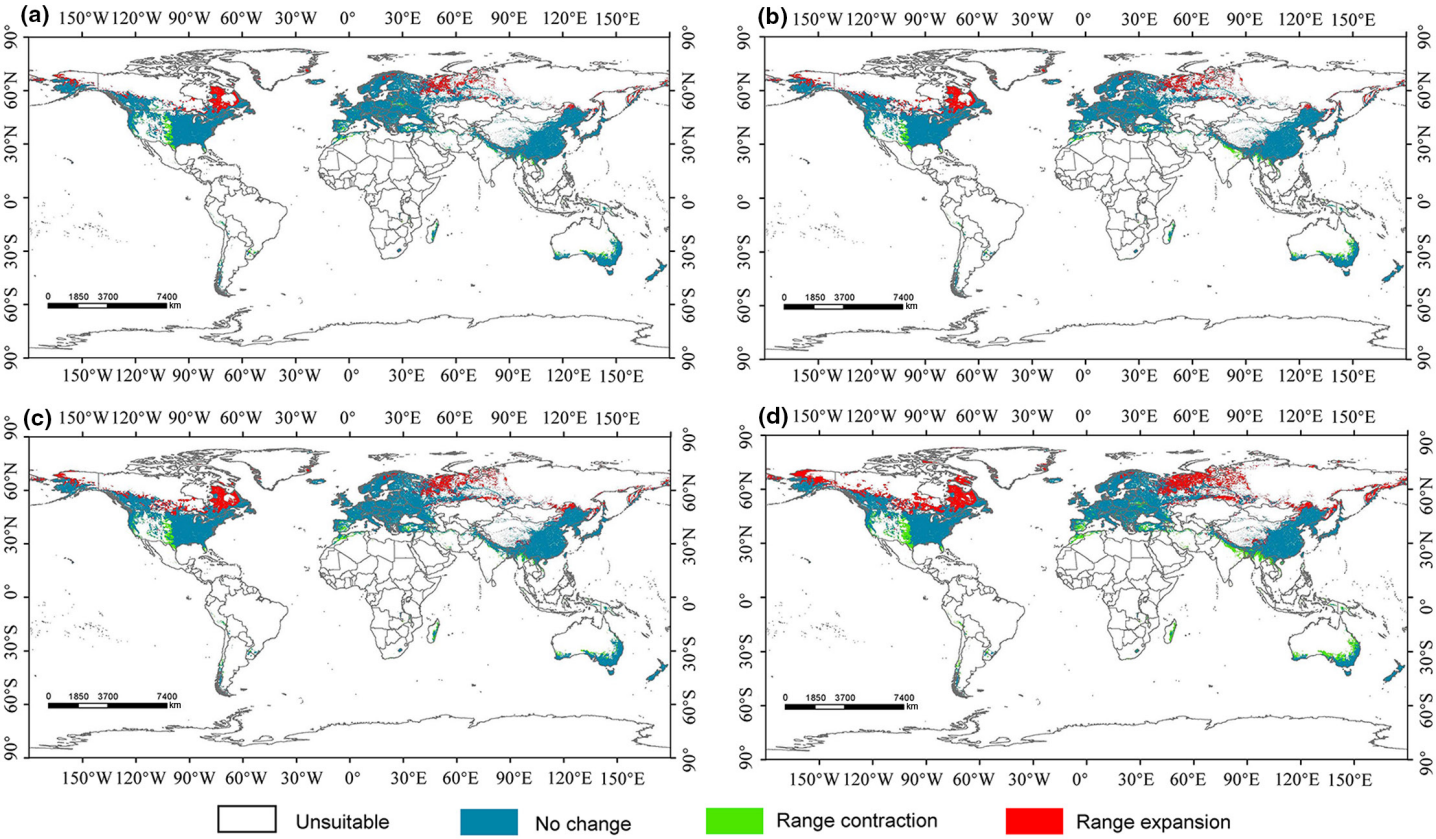
Future area changes in different suitable areas for *Cenococcum geophilum* relative to current climate change scenarios (a: 2050 SSP 1–2.6; b: 2070 SSP 1–2.6; c: 2050 SSP 5–8.5; d: 2070 SSP 5–8.5). The red color represents expansion areas, green represents contraction areas, blue represents unchanged areas, and white represents unsuitable areas.

To investigate the effects of climate change on *C. geophilum* habitats, we utilized four scenarios (two SSP scenarios for the 2050s and 2070s, respectively) to model habitat suitability (Figure 5). Under the SSP1‐2.6 scenario, characterized by the lowest greenhouse gas (GHG) emissions, suitable habitats for *C. geophilum* expanded by 9.21% in the 2050s and 9.97% in the 2070s compared to the current time (Figure 6). This expansion trend intensified in the SSP5‐8.5 scenario, representing the highest GHG emissions, with a 15.10% increase in suitable habitats for *C. geophilum* in the 2050s and a 21.02% increase in the 2070s compared to the current period (Figure 6). Therefore, the area of suitable habitat for *C. geophilum* increases with time and the extent of greenhouse gas emissions. Comparative analyses of changes in spatial patterns of *C. geophilum* under two different future climate scenarios suggest that climate change will lead to an expansion of habitat for *C. geophilum*, and that this expansion will occur mainly in the northern regions, particularly in Russia (north of Kazakhstan, the eastern coast), southern and eastern Canada, northwestern and central Alaska, and the United States (Figure 7).

## 4 DISCUSSION

### 4.1 Accuracy of MaxEnt after optimization

In this study, we employed the optimized MaxEnt model to analyze the global distribution of *C. geophilum* over time within the context of global climate change. Although the MaxEnt model is widely used for simulating species' potential distribution under climate change due to its stability and accuracy, previous studies have demonstrated that using default parameters may lead to overfitting and sampling bias, thereby affecting prediction accuracy (Li et al., 2023; Phillips et al., 2006; Shi et al., 2023). Therefore, in this study, we optimized the MaxEnt model using the kuenm package, which identifies the optimal combination of model parameters by iteratively refining the fit between distributional records and environmental variables (Cobos et al., 2019). By adjusting the model parameters and comparing the outcomes, we found that optimizing the RM value from 1 to 4 and the FC value LQPH to QPH reduced the delta AICc of the model from 504.4341 to 0 and therefore the overfitting of the model is greatly reduced (Table 1). The AUC value of the optimized model is 0.878 and the TSS value is 0.959, indicating that the model has a high prediction accuracy (Table S3, Figure S2). The findings reinforce the importance of model optimization, consistent with previous studies (Ma et al., 2023).

### 4.2 Important environmental factors affecting the distribution of *C. geophilum*

According to the percent contribution, permutation importance, and the jackknife test of the MaxEnt model, we have concluded that the mean temperature of the coldest quarter (−17.92 to 17.42°C), annual precipitation (≥280.73 mm), temperature seasonality (188.54%–1548.8%), precipitation of the driest quarter (≥4.87 mm), and topsoil electrical conductivity (≥0.034 dS/m) are the dominant factors restricting the current distribution of *C. geophilum* (Figure 2). These statistics suggest that *C. geophilum* is better suited to areas that are cooler, wetter, and have four distinct seasons. These findings are consistent with previous studies indicating that *C. geophilum* dominates temperate to boreal forest systems (Obase et al., 2016).

Most of the important variables related to *C. geophilum* survival are closely associated with climatic factors, particularly temperature and precipitation. Temperature has a greater relative importance compared with precipitation. A recent global meta‐analysis on mycorrhizal fungi also suggests that temperature and precipitation, especially warming, are strong predictors of climate change impacts on ECM fungi (Bennett & Classen, 2020). In terms of climate preferences, *C. geophilum* not only favors moderately cold climates but also exhibits physiological adaptations to extreme cold. The survival of *C. geophilum* in the Arctic further supports this observation (Miyamoto et al., 2022). Additionally, *C. geophilum* can thrive even under relatively arid conditions, potentially due to its ability to regulate osmotic pressure, and enhance antioxidant enzyme activity and alleviate drought stress (Li, Yuan, et al., 2022).

Topsoil electrical conductivity, a soil factor, also influences the distribution of *C. geophilum*, but its importance is lower compared with climate factors. Interestingly, some studies have identified soil factors such as temperature, humidity, and pH as environmental filters strongly affecting ECM communities (Ge et al., 2017; Kwatcho Kengdo et al., 2022). Although soil factors have a strong influence at smaller scales, they may not drive the global distribution patterns of ECM fungi. Therefore, we argue that climate warming will alter the suitable habitat for *C. geophilum*, with temperature and precipitation as the dominant variables that determine its potential global distribution.

### 4.3 Response of the spatial distribution pattern of *C. geophilum* to climate change

The potential distribution areas of *C. geophilum* currently include the northern and southern parts of China, Korea, and Japan in Asia; the west‐central part of Europe; the western seaboard and eastern regions of North America; and the southeastern part of Australia (Figure 3). These predictions are consistent with the summarized geographic distribution of *C. geophilum* by Tapper (1964).

Through modeling and predicting the range of *C. geophilum* at different time periods, we can infer the historical causes of its formation and future population dynamics. Fossil records indicate that ECM fungi were present in host roots during the Eocene epoch, approximately 48.7 Ma, and the tropics have been identified as the origin of many ECM fungal lineages (Lepage et al., 1997). Our study suggests that during the LGM, the center of distribution of *C. geophilum* was not only in the equatorial region but also in the North Temperate Zone (Figure 4). This finding supports the idea of the tropics as the origin of many ECM fungi, while also indicating that certain regions in the north temperate zone, such as the mountain ranges in southern China, may have provided diverse and stable environmental conditions for ECM fungi during the LGM period. Glacial refuges play a crucial role in the long‐term survival and dynamics of living organisms (Tzedakis et al., 2002). The complex topography and heterogeneous environments of the Qinling‐Huaihe Line region in China and the thermal stability and slow biological turnover of Peninsular Malaysia East offer significant opportunities for long‐term species formation and persistence of ECM fungi (Liu et al., 2022; Wilf & Kooyman, 2023). Both regions are projected to serve as refuges for the northward and southward movements of *C. geophilum* in response to climatic oscillations during the glacial period.

During the Mid‐Holocene period, the suitable survival zone of *C. geophilum* migrated slightly northward (Figure 4). However, the disappearance of the suitable survival zone in Peninsular Malaysia may be attributed to the tropical temperature exceeding the critical temperature necessary for ECM fungi to survive (Steig, 1999).

Differences in predictions of potential future suitable areas for *C. geophilum* have been observed across different timeframes and climate scenarios. However, the general trends consistently indicate an expansion of the suitable area for *C. geophilum* in the future (Figure 6). The expansion is primarily observed in the northern part of the current suitable area, while a contraction is predicted in the southern part (Figure 7). These findings align with previous research indicating that ECM fungi, such as *C. geophilum*, are likely to migrate toward higher latitudes as the climate continues to warm (Dahl et al., 2023). Furthermore, the expanded suitable areas for *C. geophilum* are primarily concentrated in the overlapping region of the north temperate and cold zones. It is, thus, believed that the increased temperature and precipitation in this area create favorable conditions for its survival and growth. Therefore, this region becomes particularly sensitive for *C. geophilum* in terms of adapting to climate change. To effectively conserve and manage ECM fungal responses to climate change, it is crucial to prioritize these habitats and develop appropriate conservation strategies. Additionally, under the same representative concentration pathway, the magnitude of change in the suitable area for *C. geophilum* is positively correlated with time. Moreover, the range of changes becomes more significant as the representative concentration pathway intensifies. This further indicates the sensitivity of ECM fungi to the impacts of climate change. Taken together, these studies suggest that future climate change aligns with the species characteristics and preferences of *C. geophilum* regarding temperature and precipitation. Consequently, it is expected that the suitable areas for *C. geophilum* and ECM fungi will expand to higher latitudes in the future.

## 5 CONCLUSION

In this study, we used the optimized MaxEnt model to simulate changes in the distribution pattern of *C. geophilum* from the Last Glacial Maximum into the future and explored the key environmental factors affecting its distribution. The results indicate that temperature and precipitation are important factors influencing the global distribution of *C. geophilum*. Specifically, the mean temperature of the coldest quarter and annual precipitation play a crucial role in determining its global distribution pattern. The Qinling‐Huaihe Line region of China and eastern Peninsular Malaysia are identified as possible refuges for *C. geophilum* during the glacial period. Currently, the potential distribution areas of *C. geophilum* are concentrated in eastern Asia, west‐central Europe, the western seaboard and eastern regions of North America, and southeastern Australia. To adapt to future global warming, the suitable habitat of *C. geophilum* is projected to shift towards higher latitudes, resulting in an expansion of the suitable survival area from 9.21% to 21.02%. Additionally, the intersection of the northern temperate and boreal zones may be a sensitive area for *C. geophilum* to cope with future climate warming. This study provides a theoretical basis for the conservation and biogeography of *C. geophilum* and ECM fungi globally, as well as new insights into the formation of their distribution patterns and evolutionary trends.

## AUTHOR CONTRIBUTIONS

**Yexu Zheng:** Data curation (equal); methodology (equal); software (equal); writing – original draft (equal); writing – review and editing (equal). **Chao Yuan:** Investigation (equal); resources (equal); writing – review and editing (equal). **Norihisa Matsushita:** Investigation (equal); resources (equal); writing – review and editing (equal). **Chunlan Lian:** Investigation (equal); resources (equal); writing – review and editing (equal). **Qifang Geng:** Data curation (equal); investigation (equal); resources (equal); writing – original draft (equal); writing – review and editing (equal).

## CONFLICT OF INTEREST STATEMENT

The authors declare there are no conflicts of interest.

## Supporting information


Appendix S1.

## Data Availability

We used open‐access data from the Global Biodiversity Information Facility database (GBIF, https://www.gbif.org/), WorldClim (http://worldclim.org), and Harmonized World Soil Database (HWSD, https://www.fao.org/soils‐portal/data‐hub/soil‐classification/en).
