# Supplementary material for: Analysis of the distribution pattern of the ectomycorrhizal fungus Cenococcum geophilum under climate change using the optimized MaxEnt model
Source: Ecol Evol. 2023 Sep 25;13(9):e10565. doi: 10.1002/ece3.10565 (PMC10518754; doi:10.1002/ece3.10565)
Supplement: Supplementary file 1 — Appendix S1. [file ECE3-13-e10565-s001.docx]

**Supplementary information**

Analysis of the distribution pattern of the ectomycorrhizal fungus *Cenococcum geophilum* under climate change using the optimized MaxEnt model

Table S1. Environmental variables used in this study.

| Category | Variable | Description |
| --- | --- | --- |
| Climate | Bio1 | Annual mean temperature |
|  | Bio2 | Mean diurnal range |
|  | Bio3 | Isothermality |
|  | Bio4 | Temperature seasonality |
|  | Bio5 | Max temperature of warmest month |
|  | Bio6 | Min temperature of coldest month |
|  | Bio7 | Temperature annual range |
|  | Bio8 | Mean temperature of wettest quarter |
|  | Bio9 | Mean temperature of driest quarter |
|  | Bio10 | Mean temperature of warmest quarter |
|  | Bio11 | Mean temperature of coldest quarter |
|  | Bio12 | Annual precipitation |
|  | Bio13 | Precipitation of wettest month |
|  | Bio14 | Precipitation of driest month |
|  | Bio15 | Precipitation seasonality |
|  | Bio16 | Precipitation of wettest quarter |
|  | Bio17 | Precipitation of the driest quarter |
|  | Bio18 | Precipitation of warmest quarter |
|  | Bio19 | Precipitation of coldest quarter |
| Topography | Elevation |  |
| Soil | AWC | Available water capacity |
|  | T_GRAVEL | Topsoil gravel content |
|  | T_SILT | Topsoil silt fraction |
|  | T_OC | Topsoil organic carbon |
|  | T_CEC_CLAY | Topsoil CEC (clay) |
|  | T_CACO3 | Topsoil calcium carbonate |
|  | T_CASO4 | Topsoil gypsum |
|  | T_ESP | Topsoil sodicity (ESP) |
|  | T_ECE | Topsoil electrical conductivity |
|  | T_TEXTURE | Topsoil texture |
|  | T_SAND | Topsoil sand fraction |
|  | T_CLAY | Topsoil clay fraction |
|  | T_USDA_TEX | Topsoil USDA texture classification |
|  | T_PH_H2O | Topsoil pH (H_2_O) |
|  | T_BS | Topsoil base saturation |
|  | T_TEB | Topsoil TEB |

Table S2. Contribution percentages and permutation importance values for each environmental variable.

| Variable | Description | Percent contribution |
| --- | --- | --- |
| Bio2 | Mean diurnal range | 4.5 |
| Bio4 | Temperature seasonality | 8.6 |
| Bio11 | Mean temperature of coldest quarter | 38.9 |
| Bio12 | Annual precipitation | 24.5 |
| Bio15 | Precipitation seasonality | 1.3 |
| Bio17 | Precipitation of driest quarter | 4.0 |
| Bio19 | Precipitation of coldest quarter | 1.1 |
| Elevation |  | 0.4 |
| AWC | Available water capacity | 0.8 |
| T_GRAVEL | Topsoil gravel content | 2.9 |
| T_SILT | Topsoil silt fraction | 0.7 |
| T_OC | Topsoil organic carbon | 0.4 |
| T_CEC_CLAY | Topsoil CEC (clay) | 1.5 |
| T_CACO3 | Topsoil calcium carbonate | 0.1 |
| T_CASO4 | Topsoil gypsum | 2.7 |
| T_ESP | Topsoil sodicity (ESP) | 1.0 |
| T_ECE | Topsoil electrical conductivity | 6.7 |

Table S3. Predictive performance of the model based on 10 repetitions using TSS values.

| species | TSS |
| --- | --- |
| *Cenococcum geophilum_0* | 0.969 |
| *Cenococcum geophilum_1* | 0.97 |
| *Cenococcum geophilum_2* | 0.944 |
| *Cenococcum geophilum_3* | 0.95 |
| *Cenococcum geophilum_4* | 0.968 |
| *Cenococcum geophilum_5* | 0.954 |
| *Cenococcum geophilum_6* | 0.968 |
| *Cenococcum geophilum_7* | 0.95 |
| *Cenococcum geophilum_8* | 0.965 |
| *Cenococcum geophilum_9* | 0.961 |
| average | 0.9599 |


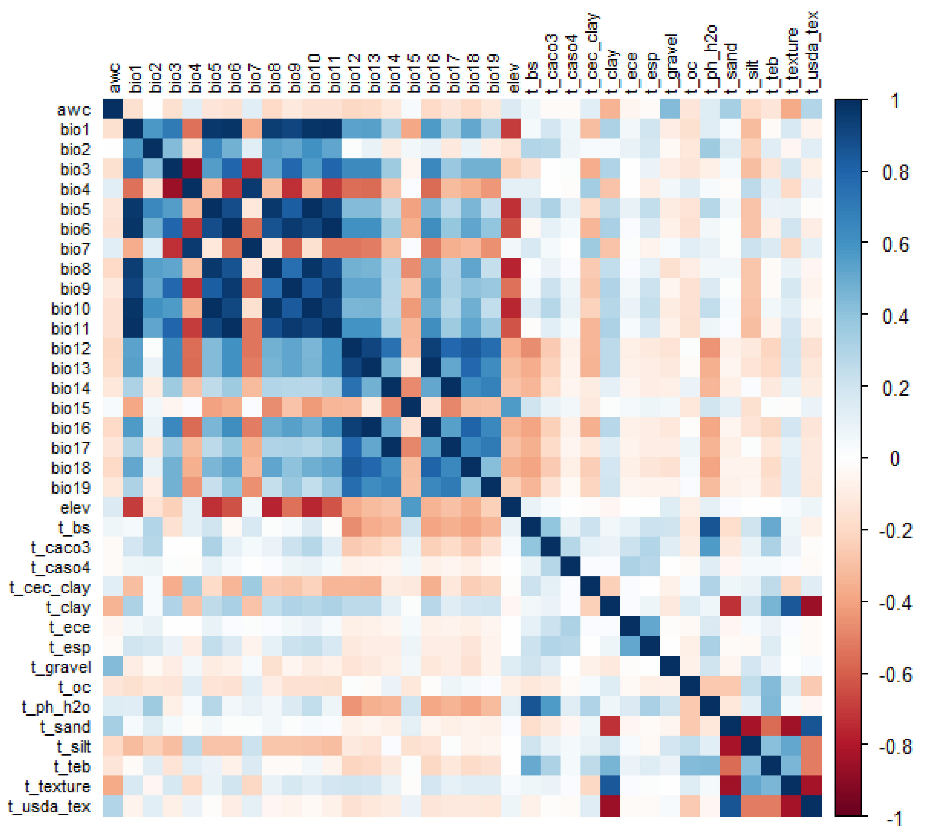


Figure S1. Correlation plot of the selected variables.


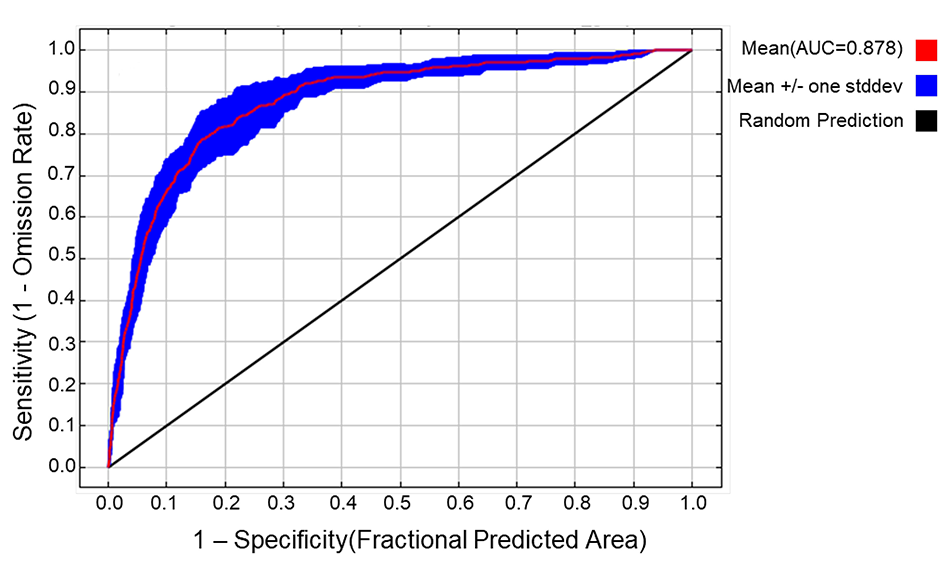


Figure S2. Receiver operating curve for training and test data with area under the ROC curve (AUC) values. The blue line represents the model fit to the testing data.


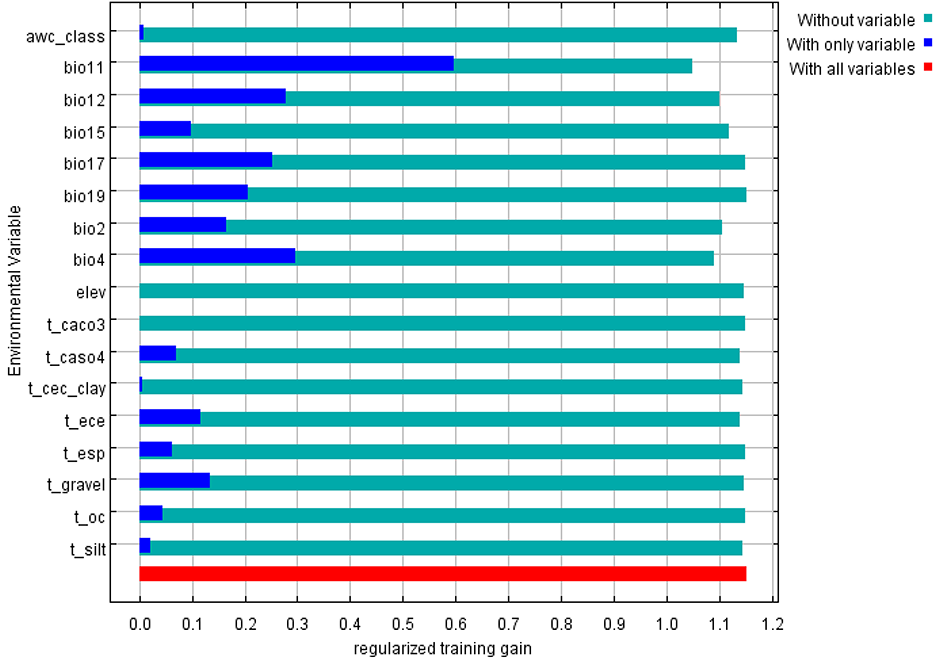


Figure S3. Jackknife test of the importance of environmental variables.
